# Supplementary material for: SARS-CoV-2 variant-specific differences in inhibiting the effects of the PKR-activated integrated stress response
Source: Virus Res. 2023 Nov 28;339:199271. doi: 10.1016/j.virusres.2023.199271 (PMC10716588; doi:10.1016/j.virusres.2023.199271)
Supplement: Supplementary file 1 [file mmc1.docx]

**Supplementary Figure 1: G3BP1 staining in ancestral SARS-CoV-2 infected Vero E6 cells.** Cells were infected with ancestral SARS-CoV-2 and fixed after 24h. Uninfected cells that were either untreated or treated with 1mM sodium arsenite (SA) were used as a control. The cells were stained with antibodies against SARS-CoV-2 N protein (magenta) and G3BP1 (green) and with DAPI (blue). Imaging was performed with confocal microscopy (20X). Scale bars 20 mm.

**Supplementary Figure 2: The effect of different stress stimuli on replication of ancestral SARS-CoV-2, Delta, and Omicron.**

(A) Effect of different stress inducers on viral replication of the ancestral strain in Vero E6 cells. SARS-CoV-2 infected cells were treated with sodium arsenite, Poly(I:C), thapsigargin or starved (see materials and methods). 6h after treatment the viral titers were determined and normalized against the titers in untreated cells. Data are represented as mean ± SEM.

n ≥ 3.

(B) Effect of different stress inducers on viral replication of ancestral SARS-CoV-2, Delta or Omicron in A549-hACE2 cells. Cells were treated with sodium arsenite, Poly(I:C), thapsigargin or starved as described in figure 2 and 6h after treatment the viral titers were determined. Data are represented as mean ± SEM. n = 3.

*, p < 0.05; **, p < 0.005; ****, p < 0.0001

**Supplementary Figure 3: Nucleocapsid protein levels in SG-negative and -positive A549-hACE2 cells infected with different SARS-CoV-2 variants.**

(A) N protein levels in individual A549-hACE2 cells. Ancestral SARS-CoV-2, Delta, or Omicron infected A549-hACE2 cells were fixed 24 hpi and stained with antibodies against SARS-CoV-2 N protein (magenta) and G3BP (green) and with DAPI (blue). Arrows show SG-positive cells. Imaging was performed with confocal microscopy (20X). Scale bars 20 mm.

(B) Quantification of N protein levels in SG-negative and -positive A549-hACE2 cells. Ancestral SARS-CoV-2, Delta, or Omicron infected A549-hACE2 cells were fixed 24 hpi and the N protein corrected total cell fluorescence (CTCF) of SG-negative and SG-positive cells was determined. For each variant, the CTCF of SG-positive cells was normalized against the CTCF of SG-negative cells. Data are represented as mean ± SEM. n = 3

**, p < 0.005

**Supplementary Figure 4: Translational levels in ancestral SARS-CoV-2 infected cells.** (A) Vero E6 cells were infected with ancestral SARS-CoV-2. Before fixation at 6 or 24 hpi, the cells were treated with puromycin. The cells were stained with antibodies against the viral N protein (magenta) and puromycin (green), and DAPI (blue). Scale bars 20 mm. (B) A549-hACE2 cells were infected with different SARS-CoV-2 variants. Before fixation at 24 hpi, the cells were treated with puromycin. The cells were stained with antibodies against the viral N protein (magenta) and puromycin (green), and DAPI (blue). Scale bars 20 mm.

**Supplementary Figure 5: The effect of ISR activation during SARS-CoV-2 infection on global translational levels and SG formation**

(A) Puromycin incorporation in ancestral SARS-CoV-2 infected Vero E6 cells. Uninfected and infected cells were treated with sodium arsenite (SA) and/or ISRIB (I). Before sample collection at 24 hpi, the cells were treated with puromycin to visualize translational levels.

(B) Quantification of (A). ﻿Puromycin levels were normalized to cellular levels of b-actin. The data are presented as the fold change in relation to the puromycin levels of untreated, uninfected cells. Data are represented as mean ± SEM. n ≥ 3.

(C) ISRIB treatment reduces SG formation. Ancestral strain-infected Vero E6 cells were treated with ISRIB and fixed at 6 hpi. They were stained for SARS-CoV-2 N protein and G3BP. For the analysis using fluorescence microscopy, ≥5 images of a total of >100 cells were taken at random positions and the number of infected cells showing SGs was determined. Data are represented as mean ± SEM. n = 3.

*, p < 0.05; **, p < 0.005

**Supplementary Figure 1**

**
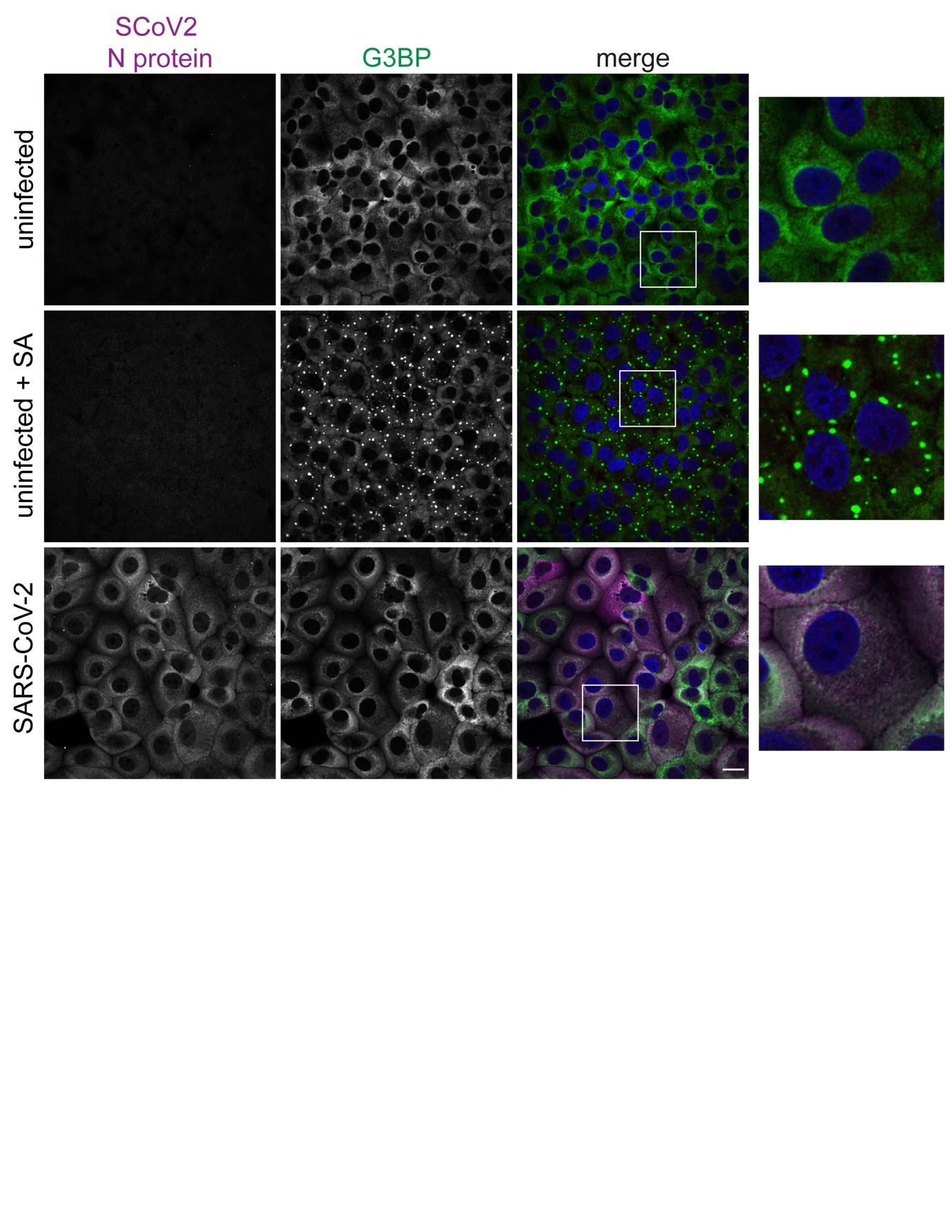
**

**Supplementary Figure 2**

**
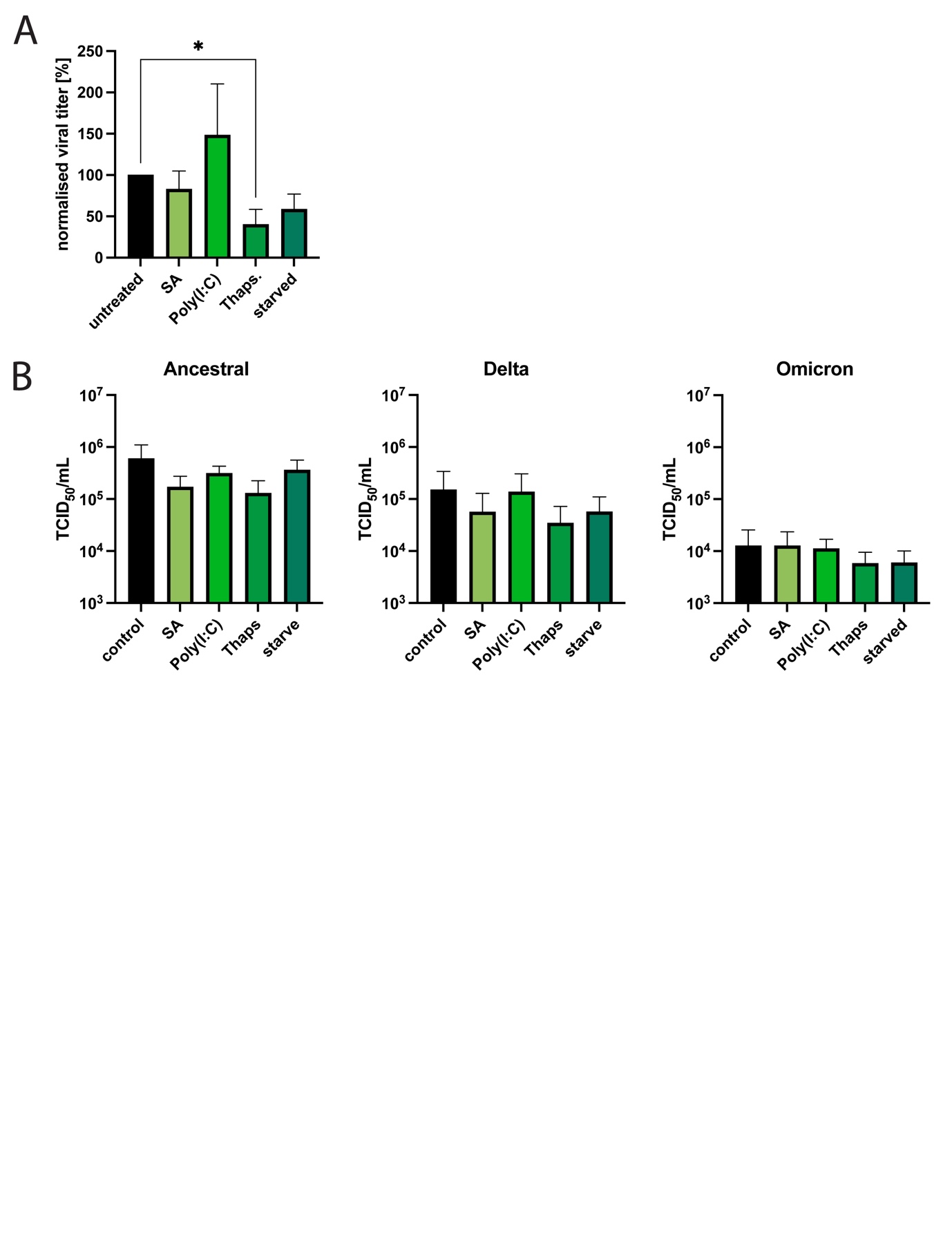
**

**Supplementary Figure 3**

**
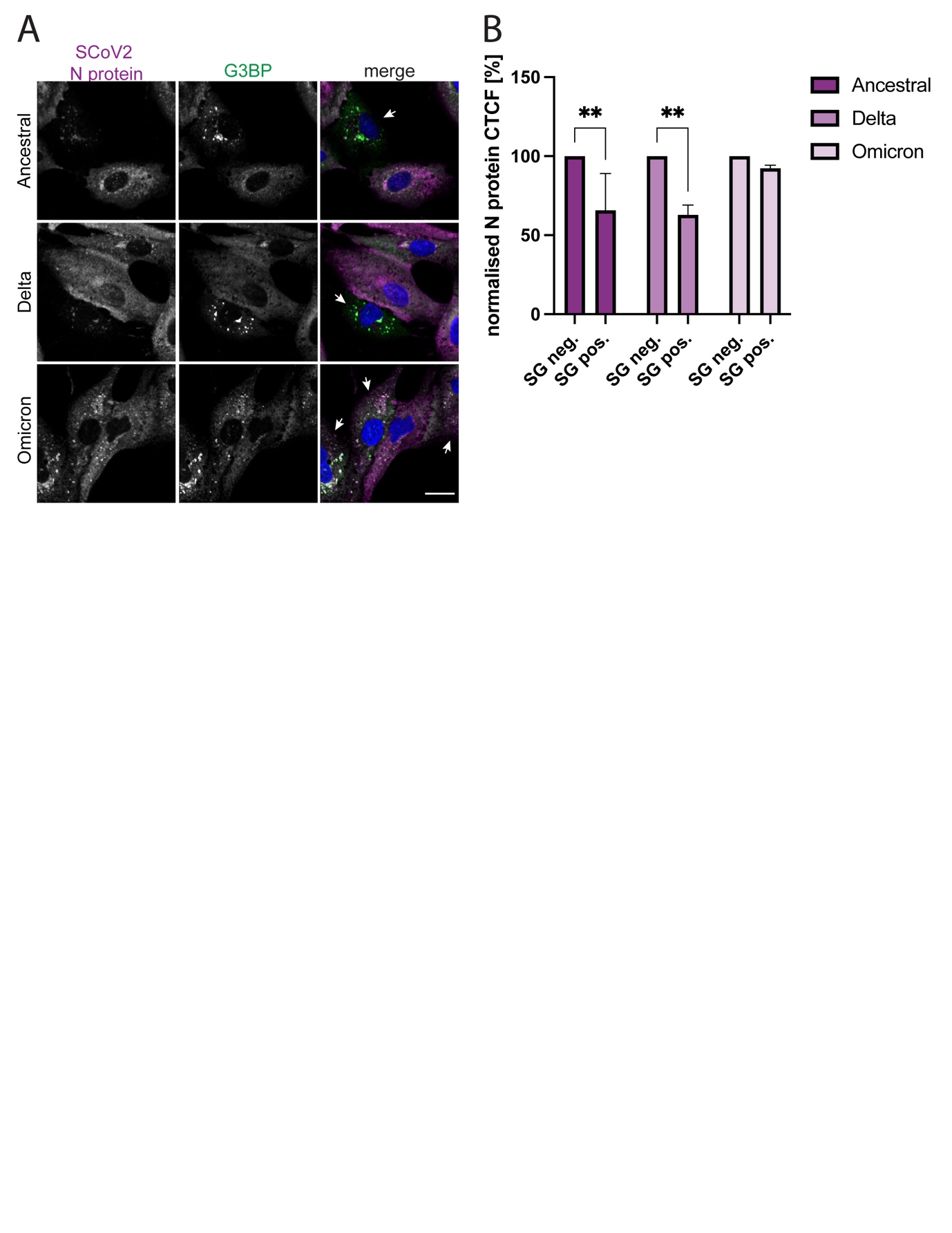
**

**Supplementary Figure 4**

**
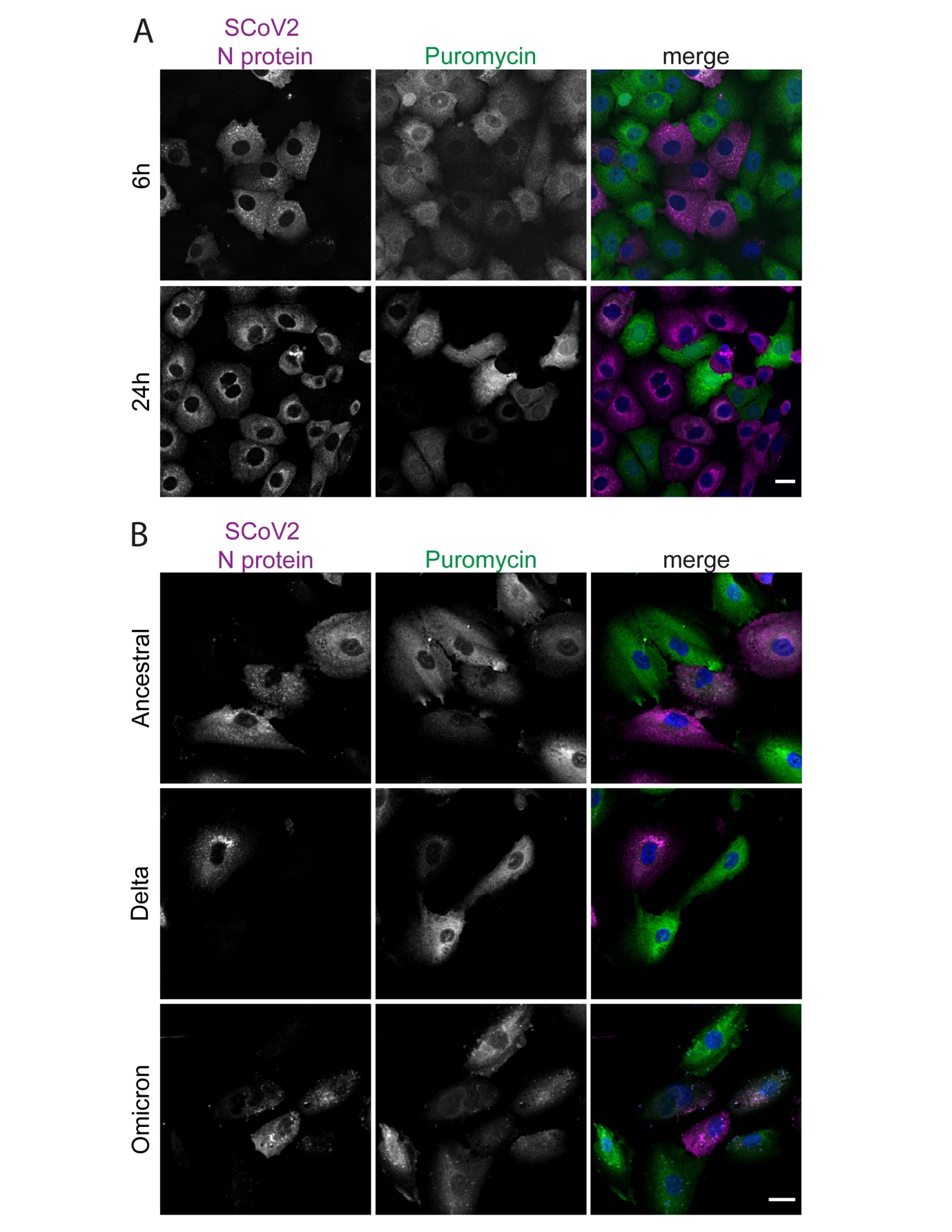
**

**Supplementary Figure 5**

**
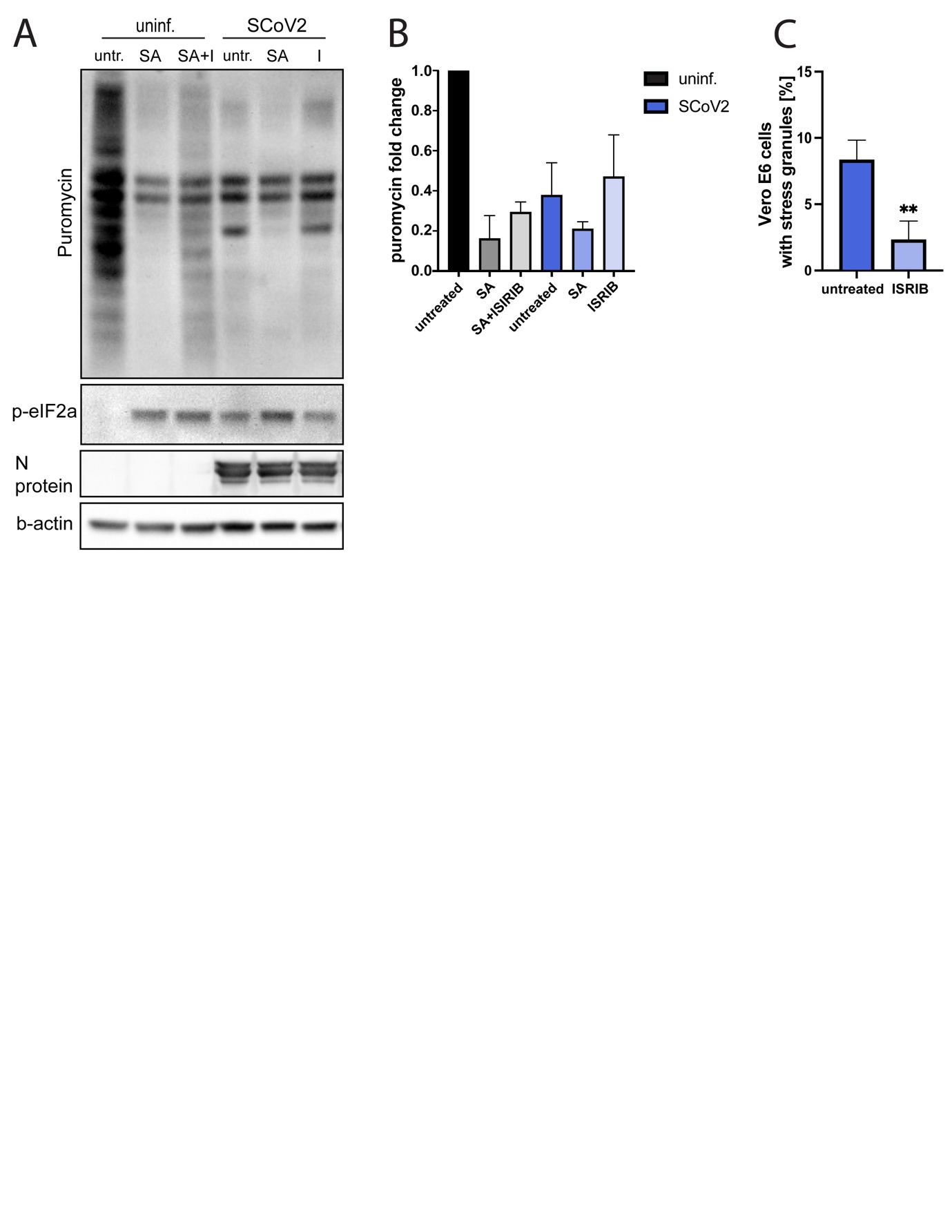
**
